# Postnatal care utilization and associated factors among women of reproductive age Group in Halaba Kulito Town, Southern Ethiopia

**DOI:** 10.1186/s13690-018-0256-6

**Published:** 2018-02-08

**Authors:** Teshome Abuka Abebo, Dawit Jember Tesfaye

**Affiliations:** School of Public and Environmental Health, Hawassa University College of Medicine and Health Sciences, Hawassa, Ethiopia

**Keywords:** Postnatal care, Reproductive age, Women

## Abstract

**Background:**

Despite postnatal care services significant role in improving maternal and new-born health, services are underutilized in most developing countries including Ethiopia. Hence, it is important to identify factors that facilitate or impede postnatal care services utilization. The aim of this study was to assess postnatal care services utilization and associated factors among reproductive age women who gave live birth in 2015 at Halaba kulito town, Southern Ethiopia.

**Methods:**

A community-based cross-sectional study was conducted on 401 reproductive age women who gave live birth a year prior to the survey. Data were collected by using structured questionnaire. Bivariate and multivariable logistic regression analysis were carried out to identify factors associated with postnatal care services utilization. A significant association was declared when *p*-value is less than 0.05. The strength of association was determined by calculating odds ratio at 95% confidence interval.

**Result:**

In this study, postnatal care services utilization by reproductive age women was 47.9%. Multivariable analysis revealed that government employed (AOR = 3.01, 95%CI = 1.36, 6.67), have three ANC visits (AOR = 4.29, 95% CI = 1.59, 11.55), have four ANC visits (AOR = 9.55, 95% CI = (3.46, 26.39), gave last birth at Health Centre (AOR = 10.76, 95% CI = 3.26, 35.57), gave last birth at Hospital (AOR = 13.15, 95% CI = (3.64, 47.50), didn’t aware of at least one postpartum danger signs (AOR = 0.06, 95% CI = (0.01, 0.37), didn’t know child care and had three ANC visits (AOR =0 .14, 95% CI (0.02, 0.8), and didn’t know child care and had four or more ANC visits (AOR =0 .13, 95% CI (0.02, 0.79) were significantly associated with postnatal care services utilization.

**Conclusion:**

This study assessed PNC services utilization and associated factors among reproductive age women. The study results provided a basic understanding of factors that associated with PNC services utilization by reproductive age women. The findings of this study showed direct association between postnatal care utilization and maternal employment, awareness to postpartum danger signs, frequency of ANC and attending birth at health institution. Therefore, the results suggested context-specific evidence which might be taken into consideration when rethinking policies to increase PNC utilization.

## Background

Postnatal period is the time period from birth to 42 days after birth. It is a period where most of the maternal and new-born death occur. Immediately after birth, bleeding and infection pose the greatest risk to the mother’s life, while preterm birth, asphyxia, and severe infections pose the greatest risk to newborn [1, 2]. Most maternal and newborn deaths are avoidable because healthcare solutions to prevent or manage complications related to pregnancy and birth are well known. All these maternal and neonatal problems could be reduced if women receive appropriate postnatal care [1].

Postnatal care (PNC) is one of the recommended strategies to reduce the maternal and new-born deaths during the postpartum period [3, 4]. Hence, World Health Organization (WHO) recommends mothers and newborns should receive PNC in health facilities for at least 24 h after birth, if birth is in a health facility. While, if birth is at home, the first postnatal contact should be as early as possible within 24 h of birth. At least three additional postnatal contacts are recommended for all mothers and newborns on day 3 (48–72), between days 7–14 after birth, and six weeks after birth [4]. However, In Africa, most of mothers and newborns did not visit the health institution following birth, indicating that postnatal care programs are among the weakest of all reproductive and child health programs [5].

In Ethiopia, the level of postnatal care coverage is extremely low. A 2011 Ethiopian Demographic and Health survey (EDHS) revealed that great majority of women (92%) with a live birth in the preceding five years did not receive a postnatal check-up. Also, it pointed out that there was regional variation in PNC utilization. Four instance in the Southern Nations, Nationality and Peoples Region (SNNPR), reported that the percentage of women had a postnatal check-up in the first two days after they gave birth was only 5.4% whereas in Addis Ababa was 47.7% [6]. Similarly, other studies conducted in different parts of Ethiopia revealed that PNC utilization by reproductive age women is low [7–13]. Meanwhile, in the Health Sector Transformation Plan (HSTP) 2015/16 Ethiopian government set a target of 95% postnatal coverage by the year 2020 [12].

Evidence showed that PNC services utilization is influenced by factors such as; maternal age, educational level of the women, occupational status of women and husbands, place of delivery, mode of delivery, the number of pregnancies, awareness about obstetric related danger sign, and awareness about PNC services [7–21].

However, factors influencing PNC services utilization vary from place to place in relation to culture and socioeconomic status of given society. Thus, assessing factors influencing postnatal care utilization in the study area is important to design public health intervention to improve PNC utilization. So that, this study was aimed to assess postnatal care utilization and associated factors among women who gave live birth in 2015 in Halaba Kulito town, Southern Ethiopia.

## Methods

### Study design, setting, and population

A community-based cross-sectional study was conducted from May 1 to 30, 2016 in Halaba kulito town. The town is administrative centre of Halaba special district which is found in Southern Nation Nationality and Peoples Region (SNNPR). It is located 245 km south from Addis Ababa through Worabe district and 90 km from Hawassa, the capital city of SNNPR. The town has five kebeles (smallest administrative units in Ethiopia). The estimated total population residing in the town is 39,507 people, with consisting of males 19,358 (49%) and females 20,149 (51%) of the population. Reproductive age group women account 9205 (23.2%) of total female population. Population density of the town is 4 persons per hectare and the average family size is estimated to be 6. In the town there are one health centre and one primary hospital. Basic essential obstetric care is provided in the health centre while comprehensive essential obstetric care is provided in the primary hospital. All reproductive age women who gave live birth in 2015 and resident of the town were included in the study population. Those women who had difficulty in communication due to severe illness were excluded from the study.

### Sample size determination and procedure

The sample size for PNC utilization was computed by using single population proportion formula by assuming that proportion of PNC utilization was 0.2 [8], 95% confidence interval, 4% margin of error and considering 10% of non-response rate. The final sample size was 422. Whereas double population formula was used to compute sample size to identify factors associated with PNC use. The computation was made via StatCalc application of Epi. Info version 7 with impute of 95% confidence interval, 80% power and case to control ratio 1:1. Odds ratio and expected prevalence of exposure among non-users were taken from studies previously conducted in the country [6, 9, 28]. Some of the exposure variables used to compute sample size were new at least one post postpartum danger sign (*N* = 214), ANC use (*N* = 236), and place of delivery (*N* = 206). Maximum sample size obtained was (total *N* = 367). However, sample size calculated using single population formula was judged sufficient source to identify associated factors with PNC services utilization.

Five kebeles found in the town were included in the study. Then after, total sample size was proportionally allocated to each kebele and systematic random sampling was used to select households where eligible women were living. First household was selected by lottery methods and then the remaining households were selected by skipping K-intervals. The units of analysis for this study were women (aged 15–49 years) who gave live birth in 2015.

### Definition of operational terms

#### Postnatal care services utilization

Women and newborns have at least one check-up by the skilled health professional within 42 days after birth at the health facility.

#### Postpartum danger sign awareness

If mother mentions at least one postpartum complication of mother and newborn occur after birth such as vaginal bleeding, fever, edema, unable to suck, vomiting everything etc.… coded 1 (Yes) and if not coded 0 (No).

#### Postnatal care awareness

If the mother mention at least one service from postnatal care services (counselling on breastfeeding, child care, immunization, family planning etc.…) coded 1 (Yes) and if not coded 0 (No).

#### Antenatal care utilization

Women have at least one check-up by the skilled health professional during her last pregnancy period.

#### Knew child care

If mother mentions at least one among child cares coded 1 (Yes) and if not coded 0 (No).

#### Reproductive age women

women whose age is between 15 and 49 years.

### Data collection and quality assurance

Data were collected using structured, closed ended interviewer administered questionnaire. The questionnaire was prepared in English and translated to Amharic. The dependent variable was PNC service utilization; and independent variables included in the questionnaire were socio-demographic characteristics, obstetric history, awareness about postpartum danger signs, ANC utilization, place of delivery and awareness about PNC service. Before actual data collection started two days training was given for data collectors on the objective, sampling procedure, and process of data collection by the principal investigators. The questionnaire was pre-tested on 10% of the calculated sample size. Additional adjustments in the sequence and wording of the questionnaire were made based on the results of the pre-test. Five BSC in public health participated in data collection through home visit. The collected data were checked daily for completeness and consistency by principal investigators.

### Data processing and analysis

Data entry, cleaning, and analysis were done using SPSS version 20.0 software. Binary logistic regression was used to identify independent effect of each independent variables on the outcome variables and multivariable logistic regression analysis used to control confounders and identify factor associated with PNC services utilization. The “Backward: conditional” method was used to perform the multivariable analyses. Interactions were explored and interaction was observed. Absence of multicollinearity among independent variables was checked. Variable whose variance inflation factor (VIF) greater than 10 were not entered to multivariable analysis [31] Appendix 1. The fitness of logistic regression models was assessed using the Hosmer-Lemeshow test. In bivariate and multivariable logistics regression analysis, a significant association was declared when the *p*-value was less than 0.05 and strength of association was determined by computing odds ratio at 95% CI.

### Ethical consideration

Support letter was obtained from Hawassa University College of Medicine and Health Sciences Ethical review board. Permission to proceed the study was attained from the town health office administrative officials. Verbal consent was obtained from the study participants after the brief explanation given on the objectives as well as the benefit of the study. Confidentiality and privacy of every respondent’s information were ensured by not using any identifiers of the study participants.

## Result

In this study, four hundred one women were interviewed from four hundred twenty two with a response rate of 95%. More than half, 246 (61.3%) of the participant were found between the age of 18 to 29. Regarding religion, the majority of respondents were Muslim 221 (55.1%) followed by Orthodox 120 (29.9%). Majority 357 (89.0%) of respondents were married. Concerning the educational level of mothers, 63 (15.7%) of the respondents were illiterate, 129 (32.2%) attended primary education and 150 (37.4%) attended junior secondary to high school education. A husband who attended college and above were 126 (31.4%) whereas who attended junior secondary to high school education were 167 (41.7%). Regarding occupation 152 (37.9%) of the mother were a housewife, 68 (17.0%) were government employed and 145 (36.2%) were self-employed. The majority of husband 201 (50.1%) were a merchant. Households which had a monthly income of < 500 Ethiopia Birr (ETB) were 51 (12.7%) while 129 (32.2%) households had a monthly income between 1501 and 2500 ETB and 99(24.7%) households had a monthly income of 2501 ETB and above (Table 1).

**Table 1:** Postnatal care utilization by socio-demographic characteristics of reproductive age women at Halaba Kulito town, Southern, Ethiopia, 2016

Characters	PNC utilized (*N* = 191)		Total	COR	95%	CI
	Yes	No	No (%)			
Age at pregnancy in years						
≤ 18 years	26	34	60 (15.0)	0.76	0.41	1.44
19–29	110	121	231 (57.6)	0.91	0.58	1.43
30–49	55	55	110 (27.4)	1		
Current age in years						
≤ 18	11	6	17 (4.2)	0.50	0.17	1.42
19–29	133	113	246 (61.3)	0.77	0.51	1.18
30–49	66	72	138 (34.4)	1		
Marital statues						
Single	3	9	12 (3.0)	0.33	0.08	1.46
Married	172	185	357 (89.0)	0.93	0.45	1.91
Divorced	16	16	32 (8)	1		
Religion						
Orthodox	61	59	120 (29.9)	0.84	0.45	1.57
Muslim	97	124	221 (55.1)	0.64	0.36	1.13
Protestant	33	27	60 (15)	1		
Place of residence						
Urban	197	182	379 (95)	1.33	0.56	3.19
Rural	13	9	22 (5.0)	1		
Maternal education						
Unable to read and write	31	32	55 (15.7)	0.42	0.20	0.89 ٭
Primary education	53	76	129 (32.2)	0.31	0.16	0.59٭
Junior to high school	66	84	150 (37.4)	0.34	0.18	0.65 ٭
College and above	41	18	59 (14 .7)	1		
Maternal occupation						
Trader	55	90	145 (36.2)	0.93	0.43	1.97
Private employed	13	23	36 (9.0)	2.00	3.66	6.77 ٭
Government employed	47	21	68 (17.0)	1.64	1.03	2.59 ٭
Housewife	76	76	152 (37.9)	1		
Paternal education						
Unable to read and write	10	8	18 (4.5)	0.69	0.25	1.89
Primary education	39	51	90 (22.4)	0.42	0.24	0.74 ٭
Junior to high school	61	106	167 (41.6)	0.32	0.19	0.52 ٭
College and above	81	45	126 (31.4)	1		
Paternal occupation						
Unemployed	1	6	7 (1.7)	0.11	0.01	0.95٭
Trader	82	119	201 (50.1)	0.46	0.29	0.72٭
Private employed	27	31	58 (14.5)	0.58	0.31	1.08
Government employed	81	54	135 (33.7)	1		
Household monthly income						
Below 500ETB	13	38	51 (12.7)	0.22	0.11	0.47٭
500-1500ETB	48	74	122 (30.4)	0.42	0.24	0.73 ٭
1500-2500ETB	70	59	129 (32.2)	0.77	0.45	1.31
Above 2501ETB	60	39	99 (24.7)	1		

* Represents significant association

### Obstetric characteristics, and postnatal care utilization

One hundred sixty-five (41.1%) respondents were pregnant for three and more times. One hundred forty-one (35.5%) respondents had three and more alive children. The majority of respondents 362 (90.3%) attended ANC during their last pregnancy. Among ANC attendants 111 (30.7%) attended for four and more times. Respondents who gave their last birth at the health center and hospital were 277 (69.1%) and 85 (21.2%) (Table 2).

**Table 2:** Postnatal care utilization by Obstetric characteristics in Halaba Kulito town, Southern, Ethiopia, 2016

Factors	PNC utilized (N = 191)			COR	95%	CI
	Yes	No	Total N (%)			
Number of pregnancy						
Only one	36	53	89 (22.2)	1		
Two times	72	75	147 (36.7)	1.41	0.83	2.40
Three and more	82	83	165 (41.1)	1.49	0.88	2.51
Number of live birth						
One	38	56	94 (23.4)	1		
Two	78	77	155 (38.7)	1.49	0.88,	2.50
Three and more	75	77	152 (37.9)	1.43	0.85	2.41
Total number of children alive						
One	42	63	105 (26.2)	1		
Two	77	78	155 (38.7)	1.48	0.89	2.44
Three and more	72	69	141 (35.2)	1.56	0.94	2.61
Had ANC						
Yes	185	177	362 (90.3)	1		
No	6	33	39 (9.7)	.174	0.07	0.42٭
Frequency of ANC						
One time	14	34	48 (13.3)	1		
Two times	34	53	87 (24.0)	1.56	0.73	3.32
Three times	57	59	116 (32.0)	2.34	1.14	4.83٭
Four times and above	80	32	111 (30.7)	6.27	2.97	13.2٭
Place of delivery						
Home	7	32	39 (9.5)	1		
Health center	130	147	277 (69.1)	4.0	1.72	9.47٭
Hospital	54	31	85 (21.2)	7.9	3.14	20.17٭
Aware of at least one danger signs of postpartum						
Yes	188	183	371 (92.5)	1		
No	3	27	30 (7.5)	0.16	0.02	1.32
Source of information						
Health professionals	155	113	268 (72.2)	1		
Friends, relatives	10	32	42 (11.3)	0.23	0.11	0.48 ٭
HEWs	15	22	37 (10.0)	0.49	0.25	1.00
Radio, television	8	16	24 (6.5)	0.36	0.15	0.88) ٭
Knew child care						
Yes	154	127	281 (70.1)	1		
No	37	83	120 (29.9)	0.37	0.23,	0.57) ٭
Who making decision						
Wife	13	9	22 (5.4)	1		
Husband	8	6	14 (3.4)	1.65	0.69	3.97
Both	170	195	365 (91.0)	1.53	0.52	4.49

* Represents significant association

Overall PNC utilization among reproductive age women was 47.9% (95%CI = (0.43, 0.53). The first within 24 h after birth and the last 42 days after birth visits of PNC were more utilized. Husband and wife jointly made a decision in 91% cases to utilize PNC. Majority 371(92.5%) of respondents aware of at least one danger signs of postpartum. Health professional were leading sources of information 268 (72.2%). More than half (56.9%) of mothers knew at least four PNC services (Table 2).

### Factors associated with PNC utilization

At bivariate analysis socio-demographic factors associated with PNC utilization were maternal education, maternal occupation, paternal education, paternal occupation, and household monthly income (Table 1). Likewise, factors such as frequency of ANC, aware of at least one postpartum danger sign, and place of delivery were associated with PNC utilization (Table 2).

Interactions were checked for variables found at the final model in multivariable analysis Appendix 2. Among interaction terms created, only knew child care by frequency of ANC had significant effect with PNC services utilization. Hence, interaction term, knew child care by frequency of ANC, was included in the final model. Consequently, factors associated with PNC utilization were maternal occupation, frequency of ANC, place of delivery, aware of at least one postpartum danger sign and knew child care by frequency of ANC. The government employed were 3 times (AOR = 3.01, 95% CI (1.36, 6.67) more likely to utilize PNC as compared to traders. Mothers who had three ANC visits were 4 times (AOR = 4.29, 95% CI = 1.59, 11.55) and who had four ANC visits were 9 times (AOR = 9.55, 95% CI = (3.46, 26.36) more likely to utilize PNC as compared to mothers who had single ANC visit. Mothers from a household where husband alone is a responsible to make decision to use PNC were 3 times more likely to use PNC as compared to a mothers from a household where mother makes decision autonomously. However, decision made by husband alone was not significantly associated with PNC utilization (AOR = 3.32, 95% CI = 0.4, 27.33). Mothers who gave last birth at Health centre were 10.76 times (AOR = 10.76, 95% CI = 3.26, 35.57) and at Hospital were 13 times (AOR = 13.15, 95% CI = (3.64, 47.50) more likely to utilize PNC as compared to mothers who gave their last birth at home. Odds of PNC utilization among reproductive age women who didn’t aware for at least one danger signs of postpartum were .06 times (AOR = 0.06, 95% CI = (0.01, 0.37) less likely to utilize PNC as compared to women who aware at least one postpartum danger signs. Mothers who didn’t know child care and had three ANC visits were 0.14 times (AOR = 0.13, 95% CI = 0.02, 0.8) less likely to utilize PNC services as compared to mothers knew child care and had one ANC visit (Table 3).

**Table 3:** Multivariable logistic regression model identifying factors associated with postnatal care utilization among reproductive age women at Halaba Kulito town, Southern, Ethiopia, 2016

Factors	AOR	95%	CI
Maternal Occupation			
Trader	1		
Private employed	1.02	0.42	2.43
Government employed	3.01	1.36	6.67٭
Housewife	1.62	0.90	2.90
Frequency of ANC			
One time	1		
Two times	2.03	0.74	5.55
Three times	4.29	1.59	11.55٭
Four times and above	9.55	3.46	26.36٭
Place of delivery			
Home	1		
Health center	10.76	3.26	35.57٭
Hospital	13.15	3.64	47.50٭
Who make decision			
Wife	1		
Husband	3.32	0.40	27.33
Both	0.52	0.16	1.67
Aware at least one postpartum danger sign			0.37
Yes	1		
No	0.06	0.01	0.37٭
Paternal occupation			
Unemployed	1		
Trader	0.99	0.24	4.08
Private employed	0.57	0.15	2.22
Government employed	1.24	0.31	4.97
Knew child care			
Yes	1		
No	1.62	0.40	6.60
Knew child care * Frequency of ANC			
Yes by One time	1		
No by Two times	0.54	0.09	3.30
No by Three times	0.14	0.02	0.80٭
No by Four times and above	0.13	0.02	0.79٭

Hosmer and Lemeshow Test (p-value = 0.523)

* Represents significant association

## Discussion

This study aimed to assess PNC utilization and associated factors among reproductive age women. In this study, PNC utilization among reproductive age women was 47.9%. The finding was higher than the national PNC utilization 13% [6] and other locally conducted studies in Ethiopia; 11% of Abi-Adi Town in Tigray, 20.2% of Jabetine district in Amhara region [8], 34.8% of Dembecha district [7], and 33.5% of Debre Markos town [9]. Also, the finding is higher than studies conducted abroad of Ethiopia such as 25.1% of the western district of Nepal [19], 43.2% of analysis of Nepal Demographic and Health Survey 2011 [14], and 30% Pakistan [20]. But the finding is lower than 66.8% of a study conducted at Gondar Zuria district Ethiopia [10], 78.3% of Adwa town, North Ethiopia [11], and 58% of Uganda [16]. The difference is might be due to the difference in study setting and method used. This indicates regional variation in the utilization of PNC services and the need for area/context specific intervention to achieve the HSTP target of postnatal care coverage by 2020.

Interestingly in this study government employed were more likely to utilize PNC services as compared to traders. A similar result was revealed at a study conducted in Nepal [14]. In contrast, different studies conducted in Ethiopia didn’t reveal an association between occupation and PNC utilization [7–11]. The higher tendency of PNC utilization by employed mothers might be due to mothers who are involved in paid employment are more likely to be economically independent and consequently have access to services, and utilize the services when they need or as recommended by their health workers [25]. Evidence showed that having a paid job empowers mothers to utilize maternal health services [26, 29, 30].

Reproductive age women who gave their last birth at a health facility were more likely to utilize PNC services as compared to reproductive age women who gave their last birth at home. The finding is similar to study conducted in Dembacha district Ethiopia [7], Debre Markos town Ethiopia [9], Jabetine district in Amhara region Ethiopia [8], Gondar Zuria district Ethiopia [10], EDHS 2011 [6], western district of Nepal [19], analysis of Nepal Demographic and Health Survey 2011 [14], Royal king of Cambodia [22], and Bangladesh [23], which revealed that giving birth at health facilities has direct significant association with postnatal care utilization. This might be due to the fact that women who gave birth at health institution have greater opportunity to be informed and educated about types, benefits, and availabilities of PNC services and danger signs of postpartum. This contact improves PNC services seeking behavior of mothers.

In this study, reproductive age women who had three times and four and more times ANC visits during their last pregnancy were more likely to utilize PNC as compared to mothers had single ANC visit. The finding is similar to study conducted in Dembacha district Ethiopia [7], Gahanna [15], Uganda [16], western district of Nepal [19] and analysis of Nepal Demographic and Health Survey 2011 [14] which revealed that mothers who attended four or more ANC visits were more likely to utilize PNC services. Evidence showed that ANC attendance and adequate counseling of mothers is associated with increased postnatal care attendance [24]. However, our interaction terms result showed, when mother didn’t know child care, high frequency to ANC visits (three times or four or more times) had less significant effect with PNC services utilization. This finding suggests that importance of improving knowledge about child care during antenatal care visits (counselling and health education).

Awareness about postpartum danger signs was directly associated with PNC utilization. Similarly, studies conducted in Jabetine district in Amhara region [8], in Debre Markos town [9], in Hossana [27] and in Nepal [17] revealed that women who were aware at least one obstetric danger sign of pregnancy are more likely to utilize PNC as compared to the women who didn’t.

Finally, unlike other studies in Ethiopia [8, 9, 28] which revealed that educational status of the women, and husbands were determinants of PNC utilization, these factors didn’t determine PNC service utilization in the current study.

### Strength and limitation

The conclusion of this study was derived from primary data through rigours descriptive and analytic data analysis. However, the study had some limitations. Hence, interpretation of the results needs certain consideration. Because the study use cross-sectional data, the study unable to conclude definite temporal relationship between independent and dependent variables. So that, associations were looked for. Also, in this study data were collected retrospectively, this might introduce recall bias. To reduce recall bias women who gave a live birth in 2015 only were recruited for the study. Small sample size is another limitation of this study. Single population proportion formula was used to derive optimum sample size for PNC service utilization and double population proportion formula was used to derive optimum sample size to determine associated factors. However, sample size calculated using single population formula was judged sufficient source to identify associated factors with PNC services utilization.

## Conclusion

This study assessed PNC utilization and associated factors among reproductive age women. The study results provided a basic understanding of factors that associated with PNC utilization by reproductive age women. The findings of this study showed direct association between postnatal care utilization and maternal employment, awareness to postpartum danger signs, frequency of ANC and attending birth at health institution. Therefore, the results suggested context-specific evidence which might be taken into consideration when rethinking policies to increase PNC utilization.

## Appendix

### Appendix 1

**Table 4:** Table indicates multicollinearity (Variance Inflation Factors)

**Coefficients** ^**a**^										
Model		Unstandardized Coefficients		Standardized Coefficients	t	Sig.	95.0% Confidence Interval for B		Collinearity Statistics	
		B	Std. Error	Beta			Lower Bound	Upper Bound	Tolerance	VIF
	(Constant)	.434	.285		1.526	.128	−.125	.994		
	Religion	−.013	.037	−.017	−.345	.730	−.085	.059	.946	1.057
	Maternal Occupation	.035	.019	.090	1.819	.070	−.003	.072	.936	1.068
	Husband educational status	.019	.028	.047	.672	.502	−.036	.074	.467	2.143
	Husband occupation	.024	.035	.045	.695	.488	−.044	.092	.538	1.858
	Monthly income	.032	.031	.062	1.020	.309	−.030	.093	.615	1.626
1	Number of pregnancy	.076	.103	.117	.737	.462	−.127	.279	.091	10.991
	Number of live birth	−.212	.138	−.324	−1.533	.126	−.483	.060	.051	19.482
	No of children currently	.179	.102	.276	1.756	.080	−.022	.379	.093	10.802
	How many ANC visit	.112	.028	.228	4.037	.000	.057	.167	.717	1.395
	Place of delivery	.178	.051	.187	3.498	.001	.078	.279	.804	1.244
	Who making decision	−.124	.054	−.113	−2.300	.022	−.229	−.018	.952	1.050
	Knew child care	−.157	.060	−.131	−2.618	.009	−.275	−.039	.915	1.093
	Aware at least one postprt danger sign	−.396	.141	−.136	−2.801	.005	−.674	−.118	.974	1.027
	Mother’s current age	.092	.076	.097	1.204	.229	−.058	.242	.356	2.805
	Age at pregnancy	−.119	.065	−.149	−1.830	.068	−.247	.009	.346	2.889
	Maternal education	−.011	.033	−.020	−.337	.737	−.075	.053	.645	1.550

a.Dependent Variable: postnatal care utilization

### Appendix 2

**Table 5:** Model with interaction term

Variables in the Equation	B	S.E.	Wald	df	Sig.	Exp(B)	95% C.I.for EXP(B)	
							Lower	Upper
Maternal Occupation								
Trader			8.517	3	.036			
Private employed	.015	.446	.001	1	.974	1.015	.424	2.430
Government employed	1.103	.405	7.418	1	.006	3.014	1.363	6.669
Housewife	.479	.299	2.569	1	.109	1.615	.899	2.902
Frequency of ANC								
One time			25.919	3	.000			
Two times	.706	.514	1.884	1	.170	2.026	.739	5.553
Three times	1.456	.505	8.310	1	.004	4.290	1.594	11.547
Four times and above	2.257	.518	19.002	1	.000	9.554	3.463	26.356
Place of delivery								
Home			16.363	2	.000			
Health center	2.376	.610	15.175	1	.000	10.761	3.256	35.567
Hospital	2.576	.655	15.451	1	.000	13.147	3.639	47.503
Who make decision								
Wife			5.592	2	.061			
Husband	1.200	1.076	1.245	1	.265	3.320	.403	27.332
Both	−.659	.599	1.210	1	.271	.518	.160	1.673
Aware at least one postpartum danger sign								
Yes								
NO	−2.768	.909	9.271	1	.002	.063	.011	.373
Paternal occupation								
Unemployed			7.268	3	.064			
Trader	−.007	.720	.000	1	.993	.993	.242	4.075
Private employed	−.558	.692	.649	1	.420	.572	.147	2.224
Government employed	.215	.709	.092	1	.762	1.239	.309	4.973
Knew child care								
Yes								
No	.484	.716	.457	1	.499	1.623	.399	6.601
Knew child care * Frequency of ANC								
Yes by One time			7.800	3	.050			
No by Two times	−.618	.924	.447	1	.504	.539	.088	3.297
No by Three times	−1.970	.888	4.923	1	.026	.139	.024	.795
No by Four times and above	−2.056	.931	4.882	1	.027	.128	.021	.793
Constant	−2.900	1.213	5.717	1	.017	.055		

## References

[1] World Health Organization (2010). WHO technical consultation on postpartum and postnatal care.

[2] World Health Organization (1998). Postpartum Care of the Mother and Newborn: a practical guide. Volth edition.

[3] Darmstadt GL, Bhutta ZA, Cousens S, Adam T, Walker N (2005). Evidence-based, cost-effective interventons: how many newborn babies can we save?. Lancet.

[4] World Health Organization. WHO recommendations on postnatal care of mother and newborn. Geneva: World Health Organization; 2013. 24624481

[5] World Health Organization. World health statistics 2013. Geneva: World Health Organization; 2013.

[6] Central Statistical Agency [Ethiopia] and ICF International (2012). Ethiopia demographic and health survey 2011.

[7] Hordofa MA, Almaw SS, Berhanu MG, Lemiso HB (2015). Postnatal care service utilization and associated factors among women in Dembecha District, Northwest Ethiopia. Science Journal of Public Health..

[8] Workineh YG, Hailu DA (2014). Factors affecting utilization of postnatal Care Service in Amhara Region, Jabitena District, Ethiopia. Science Journal of Public Health.

[9] Limenih M, Endale Z, Dachew B. Postnatal care service utilization and associated factors among women who gave birth in the last 12 months prior to the study in Debre Markos town, Northwestern Ethiopia: a community-based cross-sectional study. Int J Reprod Med. 2016. Article ID 7095352:7. 10.1155/2016/7095352 PMC4940555 27433481

[10] Tesfahun F, Work W, Mazengiya F, Kifle M (2014). Knowledge, perception, and utilization of postnatal care of mothers in Gondar Zuria District, Ethiopia: a cross-sectional study. Maternal and ChildHealth Journal.

[11] Berhe H, Tilahun W, Aregay A, Bruh G, Gebremedhim H (2013). Utilisation and associated factors of postnatal care in Adwa town, Tigray, Ethiopia-a cross-sectional study. Advanced Research in Pharmaceuticals and Biologicals.

[12] The Federal Democratic Republic of Ethiopia Ministry of Health (2015). Health sector transformation plan.

[13] Worku G, et al. Factors affecting utilization of skilled maternal care in Northwest Ethiopia: a multilevel analysis. BMC Int Health Hum Rights. 2013;13:20 10.1186/1472-698X-13-20 PMC3639034 23587369

[14] Khanal V (2014). Factors associated with the utilization of postnatal care services among the mothers of Nepal: analysis of Nepal demographic and health survey 2011. BMC Womens Health.

[15] Doku D (2012). Factors associated with reproductive health care utilization among Ghanaian women. BMC Int Health Hum Rights.

[16] Aminah K (2006). Factors determining the utilization of postpartum care services in Uganda, UDHS.

[17] Dhakal S, et al. Utilization of postnatal care among rural women in Nepal. BMC pregnancy and child birth. 2007;7(19) 10.1186/1471-2393-7-19 PMC2075509 17767710

[18] Dhakal S, Chapman GN, Simkhada PP, Van Teijlingen ER, Stephens J, Raja AE. Utilisation of postnatal care among rural women in Nepal. BMC Pregnancy Childbirth. 2007;7:19. 10.1186/1471-2393-7-19. 10.1186/1471-2393-7-19 PMC2075509 17767710

[19] Paudel M, Khanal V, Acharya B, Adhikari M (2013). Determinants of postnatal service utilization in a Western District of Nepal: community based cross sectional study. J Women’s Health Care.

[20] Sultana N, Shaikh BT (2015). Low utilization of postnatal care: searching the window of opportunity to save mothers and newborns lives in Islamabad capital territory, Pakistan. BMC Res Notes.

[21] Nankwanga A (2004). Factors influencing utilization of postnatal services in Mulago and Mengo hospitals [master thesis], Kampala.

[22] Ith P, Dawson A, Homer CSE, Whelan AK (2013). Practices of skilled birth attendants during labor, birth and the immediate postpartum period in Cambodia. Midwifery.

[23] Mosiur Rahman M, Haque SE, Sarwar Zahan M (2011). Factors affecting the utilization of postpartum care among young mothers in Bangladesh. Health and Social Care in the Community.

[24] Rahman M, Haque S, Zahan M (2011). Factors affecting the utilization of postpartum care among young mothers in Bangladesh. Health Soc Care Comm.

[25] Chakraborty N, Islam M, Chowdhury RI, Bari W (2002). Utilisation of postnatal care in Bangladesh: evidence from a longitudinal study. Health Soc Care Comm.

[26] Salam A, Siddiqui S (2006). Socioeconomic inequalities in use of delivery care services in India. J Obstet Gynecol India.

[27] Dutamo Z (2015). Maternal health care use among married women in Hossaina, Ethiopia. BMC Health Serv Res.

[28] Tarekegn SM (2014). Determinants of maternal health service utilization in Ethiopia: analysis of the 2011 Ethiopian demographic and health survey. BMC Pregnancy and Childbirth.

[29] Rwabufigiri BN (2016). Factors associated with postnatal care utilization in Rwanda: a secondary analysis of 2010 demographic and health survey data. BMC Pregnancy and Childbirth.

[30] Jayaraman A, Chandrasekhar S, Gebreselassie T (2008). Factors affecting maternal health care seeking behavior in Rwanda. DHS working paper no. 59.

[31] Neter J, Kutner M, Wasserman W, Nachtsheim C, Neter J (2004). Applied linear regression models 4th ed.

